# When natural selection should optimize speed-accuracy trade-offs

**DOI:** 10.3389/fnins.2014.00073

**Published:** 2014-04-10

**Authors:** Angelo Pirrone, Tom Stafford, James A. R. Marshall

**Affiliations:** ^1^Department of Psychology, University of SheffieldSheffield, UK; ^2^Kroto Research Institute, University of SheffieldSheffield, UK; ^3^Department of Computer Science, University of SheffieldSheffield, UK

**Keywords:** decision-making, value, reward, error, Bayes risk, drift-diffusion, mechanism, evolution

## 1. Introduction

In psychology and neuroscience, and in other disciplines studying decision-making mechanisms, it is often assumed that optimal decision-making means statistical optimality. This is attractive because statistically optimal decision procedures are known, can be simply implemented in biologically-plausible models, and because such models have been shown to give good fits to behavioural as well as neural data. Here we question when statistical optimality is the kind of optimality we should expect natural selection to aim towards, by considering what kinds of loss function should be optimised under different behavioural scenarios. In laboratory settings subjects are often rewarded only on making a correct choice, so optimisation of a zero-one loss function is appropriate, and this is achieved by implementing a statistically-optimal decision procedure that gives the best compromise between speed and accuracy of decision-making. Many naturalistic decisions may also be described by such a loss function; however others, such as selecting food items of potentially different value, appear to be different since the animal is rewarded by the value of the item it chooses regardless of whether it was the best available. We argue that most naturalistic decisions are value-based. Mechanisms that optimise speed-accuracy trade-offs need to be parameterised, using information about the decision problem, in order to deal with value-based decision-making. Mechanisms for value-sensitive decision-making have been described, however, which adaptively change between decision-making strategies without the need for continual re-parameterisation.

## 2. Speed-accuracy trade-offs

It is usually assumed that decision-makers have to decide to be either fast or accurate. When speed is important mistakes are more frequent, while when accuracy is needed decisions are slower. This obvious problem is defined as the speed-accuracy trade-off and is a distinctive feature of many types of decision making (Wickelgren, 1977).

The speed-accuracy trade-off can be explained within the theoretical framework of sequential sampling models of decision making that have been shown to fit behavioral and neural data from human and animal choice tasks (Ratcliff and Rouder, 2000; Ratcliff et al., 2003, 2004; Ratcliff and Smith, 2004; Busemeyer et al., 2013). In particular, the Drift Diffusion Model (DDM; Ratcliff, 1978) describes choice between two alternatives (see Smith and Ratcliff, 2004; Bogacz et al., 2006; Basten et al., 2010) and recently has been shown also to be quantitatively accurate in describing trinary choices (Krajbich and Rangel, 2011) and value-based choices (Krajbich et al., 2010; Milosavljevic et al., 2010; Krajbich and Rangel, 2011; Krajbich et al., 2012), suggesting that the DDM can be thought of as a unifying computational framework for describing decision making (Basten et al., 2010). Moreover, Bogacz et al. (2006) have demonstrated that several connectionist decision-making models can approximate the DDM under specific conditions. The DDM is a special case of the statistically-optimal Sequential Probability Ratio Test (SPRT; Wald, 1947; Wald and Wolfowitz, 1948). In the DDM noisy sensory evidence supporting the alternatives is integrated over time until the net evidence in favor of one alternative exceeds a certain positive or negative threshold value, precipitating a decision for the corresponding alternative. These thresholds can be varied to compromise optimally between the average speed and accuracy of decisions.

## 3. Speed-value trade-offs

In situations where decisions are rewarded according to whether they are correct or not, optimizing the speed-accuracy trade-off is sensible. When decisions are rewarded according to the value of the option chosen, however, a different criterion needs to be optimized. This can be illustrated with the simplest case of choosing between two equal value options; here there is no decision accuracy, since choosing either option is “correct.” Similarly, there is no difference in average evidence for which of the two options is more valuable, meaning that the SPRT/DDM will only reach a decision by integrating sufficient noise to cross a decision threshold. Thus in this scenario there is no speed-accuracy trade-off to manage; the optimal decision is to choose anything as quickly as possible. The fundamental insight is that for certain decisions, speed-value trade-offs are more appropriate to optimize, rather than speed-accuracy trade-offs.

**Figure 1:**
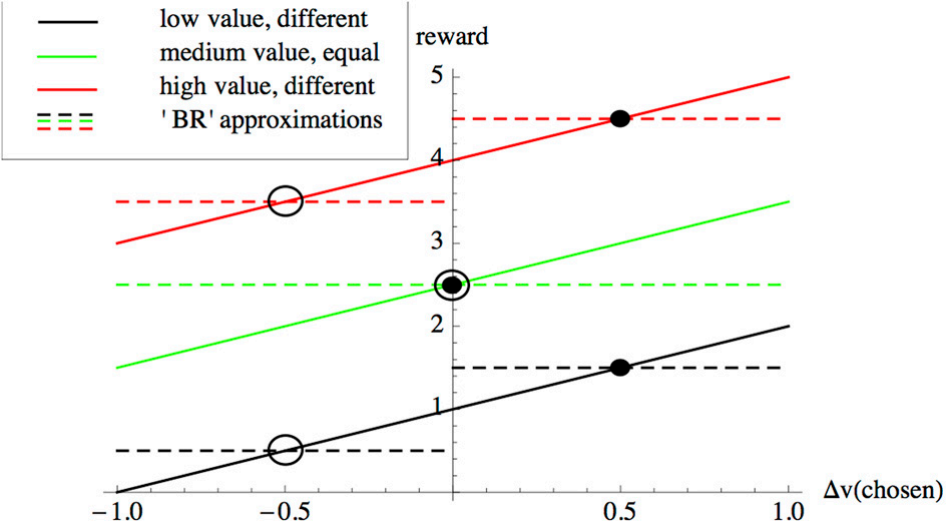
**The accuracy-based component of Bayes Risk (*BR*_*E*_ as defined by Equation 1) can be used to approximate a value-based reward scheme**. In value-based decisions individuals are rewarded according to the value |*v*| + Δ*v* of the option they choose (solid lines), where |*v*| is the average value of the alternatives under consideration, and Δ*v* is the deviation from this average of the value of the option chosen by the subject. With knowledge of the values of the alternatives, *BR*_*E*_ can be used to optimize value sensitive decision-making as described in the main text; for example the dashed lines show payoffs used in *BR*_*E*_ for: options having values of 0.5 and 1.5 units (black), options having equal values of 2.5 and 2.5 units (green) and options having values of 3.5 and 4.5 units (red). Intersections between payoffs selected for *BR*_*E*_ (dashed lines) with value-based reward (solid lines of matching colors) correspond to choice scenarios between different-valued options for which *BR*_*E*_ implements reward-by-value of the selected option; these intersections represent choice scenarios involving “poor” (hollow circles) and “good” (filled circles) options having particular values. However, the cost parameters for *BR*_*E*_ need to be recalculated according to the values of the options under consideration; for example, although the difference in the values of the alternatives does not change from the low-value (black) to the high-value (red) scenarios, since their absolute values change the *BR*_*E*_ payoffs need to be recalculated in each case. As described in the text, value-sensitive decision-mechanisms have been described that are able adaptively to deal with a variety of such decision scenarios, without re-parameterizations.

The SPRT/DDM can be optimized to take account of the value of the alternatives but, as we discuss here, doing so requires knowledge of the decision problem faced. The thresholds for an optimal decision depend on the goals of the decision maker and are task specific. By way of example, one route to accounting for the values associated with different decision outcomes is to minimize an extended version of the Bayes Risk (BR). BR is a linear combination of expected decision delay and expected terminal decision loss, first proposed by Wald and Wolfowitz (1948), and assumes that decision makers seek to minimize a cost function that is the weighted sum of decision times (DTs) and error rate (ERs). This was subsequently extended by Edwards to also account for non-zero rewards for incorrect decisions (Edwards, 1965; Bogacz et al., 2006). Formally Edwards' extension of BR, which implements Wald and Wolfowitz's version as a special case, can be defined as

(1) $$BR_{E}=c_{1}DT+c_{2}(\begin{array}{c} ER\\ 1-ER \end{array})$$

where *c*_1_ is the cost of observing the stimulus per unit time, while *c*_2_ is a row-vector specifying the payoffs from incorrect and correct choices (Bogacz et al., 2006). If *c*_2_ = (*k* 0), where *k* > 0 is a constant, then Wald and Wolfowitz's original BR is recovered. Several studies demonstrate that, under specific circumstances, subjects choose decision thresholds close to those that minimize *BR*_*E*_ (Busemeyer and Rapoport, 1988; Mozer et al., 2002). Bayes risk is not the only criterion proposed to date that decision-makers might optimize. Bogacz et al. survey alternatives, such as reward-rate, however, these alternatives are all calculated based on decision-accuracy, which requires explicit parameterizations based on the values of correct and incorrect choices (Bogacz et al., 2006). We therefore concentrate our analysis on Bayes risk. Bayes risk can be used to optimize value-sensitive decision-making; for example in a decision between two equal alternatives, each having value *v* if chosen, we would set the vector *c*_2_ = (*v v*) (e.g., dashed green line in Figure 1), thus simplifying Equation (1) above to

(2) $$BR_{E}=c_{1}DT+v.$$

Equation (2) shows us that, intuitively, an optimal decision-maker in our equal-alternatives scenario should minimize decision-time *DT*, since doing so incurs no penalty as the error rate *ER* no longer features. However, using Bayes risk in this way requires the values of the alternatives to be known on a case by case basis, as shown in Figure 1. Subjects might learn the values of incorrect and correct choices over time, for example when trials are blocked in psychophysical experiments (see Bogacz et al., 2006). However, in the following we argue that in most naturalistic decision scenarios decision-makers will not have this opportunity, and will therefore use other mechanisms that directly optimize speed-value trade-offs, rather than optimizing decisions indirectly via optimization of the speed-accuracy trade-off with an appropriate payoff vector *c*_2_.

## 4. Naturalistic decisions are usually value-based

We argue that most naturalistic decisions faced by animals, including humans, are value-based, in that the animal is rewarded according to the value of the option it chooses. Such a view on decision-making is not new to behavioral ecologists, where a long tradition exists of studying behaviors such as mate choice and foraging (Davies et al., 2012) or nest-site selection (Stroeymeyt et al., 2014). Recently many studies have focused on how value and reward are represented and integrated during the decision process (Platt and Glimcher, 1999; Sugrue et al., 2004; Padoa-Schioppa and Assad, 2006; Rangel et al., 2008; Kable and Glimcher, 2009; Krajbich et al., 2010; Philiastides et al., 2010; Hare et al., 2011; Krajbich and Rangel, 2011; Louie and Glimcher, 2012; Tsetsos et al., 2012; Cassey et al., 2013; Towal et al., 2013); however, in psychology and neuroscience, experiments are usually designed such that there is always a correct choice, and only correct choices are rewarded (see Gold and Shadlen, 2003; Bogacz et al., 2006). While studying behavior in psychophysical tasks is beneficial in that it gives a well-controlled decision environment, our point is that only rewarding subjects when they make correct choices may not correspond to the kind of decisions animals, and their neural circuitry, have typically evolved to deal with. Even in the value-based decision experiments cited above, which are analyzed using the DDM, it is typical to only present subjects with a choice between options known to have *different* values. Moreover, even though some studies have looked at how reward information is integrated (Rorie et al., 2010; Gao et al., 2011), much of this work has not yet focused on the tradeoff between value and speed. While usually in the decision-making literature the optimal behavior is to optimize speed-accuracy trade-offs, and subjects can apparently do this (Busemeyer and Rapoport, 1988; Bogacz et al., 2006), we argue that these scenarios are not representative of many naturalistic settings, and that there is great value in considering how subjects make value-sensitive decisions and how these should be optimized. In the following section we discuss theory that may be useful for this.

At least one important class of naturalistic decisions does require optimization of speed-accuracy trade-offs; these are life-or-death decisions. If we analyze for example the case of an animal attempting to forage while avoiding predators (Trimmer et al., 2008), a slow-but-accurate decision would mean being killed by the predator, a maximal loss. On the other hand if the decision is fast-but-inaccurate the animal would escape even when the stimulus is not a predator, and this would mean losing food. The best strategy for the animal is thus that which optimizes the speed-accuracy trade-off, taking into account the payoffs arising from the different decision outcomes; hence Trimmer et al.'s hypothetical animal is modeled with a single-threshold DDM, with evidence sufficient to cross that single decision threshold leading to the animal taking anti-predator action such as running away.

## 5. Mechanisms for value- sensitive decision-making

Recent modeling work inspired by studying another value-sensitive decision-making system, collective nest-site selection by honeybees (Seeley et al., 2012), has described a very simple mechanism able to adaptively account for the value of different decision outcomes, with minimal parameter tuning (Pais et al., 2013). This simple model implements a variety of sophisticated decision-making strategies; for example, when equal but low-value alternatives are presented, a decision deadlock is maintained that can be broken should a third, higher-value alternative, be made available. However, if equal-but-high-value alternatives are presented, or sufficient time passes, deadlock is spontaneously and randomly broken (Pais et al., 2013). This is particularly interesting, since the classic DDM is insensitive to the absolute value of the alternatives under consideration, and only integrates the difference in their values. When differences between alternative values are sufficient, the value-sensitive mechanism of Pais et al. becomes closer to a classic DDM, allowing speed-accuracy trade-offs to be managed, although not optimized, through modification of decision thresholds. All of the different behavioral regimes of the model arise without direct parameterizations regarding alternatives' values, simply through the dependence of the model's dynamics on the mean values of inputs to its integrator populations; this allows the model to adaptively respond to different decision scenarios on a trial-by-trial basis, which cannot be achieved in pure DDM models without the decision-maker having access to explicit information on the decision-task at hand. Modifications to DDM-type models have been proposed to deal with trial-by-trial variability such as online estimation of task parameters (Deneve, 2012) or the use of time-dependent change in parameters such as decision-thresholds, urgency signals or asymmetry of inhibition (Ditterich, 2006; Hanks et al., 2011; Drugowitsch et al., 2012; Thura et al., 2012); fundamentally, however, these modifications are still interpreted under the assumption that decision speed vs accuracy is the trade-off to be maximized, unlike the model of Pais et al. (2013) in which the dynamics are naturally interpreted in terms of value vs time trade-offs. Pais et al.'s mechanism also exhibits other characteristics of natural value-discrimination systems, such as Weber's law of just-noticeable difference; interestingly Weber's law arises from the deterministic dynamics of the mechanism rather than from noise processes (Pais et al., 2013) (cf. Deco and Rolls, 2006; Deco et al., 2007). Finally, it is important to note that the DDM cannot account for the non-linearity that characterizes many decision making dynamics (e.g., food recruitment by social insects; (Nicolis and Deneubourg, 1999) while the model of Pais et al. (2013) is non-linear.

## 6. Conclusion

The study of speed-accuracy trade-offs has been tremendously fruitful for psychology, neuroscience and animal behavior, and will doubtless prove fruitful for many years to come. Yet as we have argued here most naturalistic decisions, which animals' brains should have evolved to optimize, are value-based rather than accuracy-based. This leads us to argue that the drift-diffusion model, which optimizes speed-accuracy trade-offs, is not an ideal computational framework to describe value-based decision-making; although it has had some success in describing particular experiments on value-based decision-making, discussed in the section “Speed-Accuracy Trade-Offs,” as we have shown here the DDM requires special case-by-case parameterizations to implement true value-based decision-making. We suggest that this limits the generality of the DDM as a unifying framework for all ecologically-relevant decision-making problems. However, recent theory has presented mechanisms that can manage value-sensitive decision problems without the additional informational requirements of the DDM. At the same time, experimental and theoretical psychologists and neuroscientists have started to tackle problems of value-based decision-making. We have presented our arguments for value in terms of animal decision-making, but unicellular organisms and individual cells also make decisions (e.g., Perkins and Swain, 2009; Latty and Beekman, 2011), and value is likely to be similarly important for these. We believe that the evolutionary perspective we have presented here should motivate further research into value-sensitivity and decision-making.

## Author contributions

James A. R. Marshall conceived of the paper; James A. R. Marshall, Angelo Pirrone, and Tom Stafford discussed the material; James A. R. Marshall developed the formal argument; Angelo Pirrone and James A. R. Marshall drafted the paper and all authors approved its content.

## Funding

Angelo Pirrone is supported by the University of Sheffield Studentship Network in Neuroeconomics.

### Conflict of interest statement

The authors declare that the research was conducted in the absence of any commercial or financial relationships that could be construed as a potential conflict of interest.
